# The Role of Perfectionism and Sport Commitment on Exercise Addiction Among Hungarian Athletes

**DOI:** 10.3390/sports13070232

**Published:** 2025-07-14

**Authors:** Tamás Berki, Zsófia Daka, Andor H. Molnár

**Affiliations:** 1 Department of Physical Education Theory and Methodology, Hungarian University of Sports Science, 1123 Budapest, Hungary; 2 Physical Education and Sports Sciences, ‘Juhász Gyula’ Faculty of Education, University of Szeged, 6725 Szeged, Hungarymolnar.andor@szte.hu (A.H.M.)

**Keywords:** exercise addiction, enthusiastic commitment, constraints commitment, perfectionism, athletes

## Abstract

Exercise addiction (EA) is a maladaptive behavior characterized by excessive physical activity, often linked to negative psychological outcomes. This study investigated the relationships between perfectionism, sport commitment, and EA in a sample of 219 Hungarian athletes (M = 22.19 years). Using path analysis, we tested a model hypothesizing that adaptive and maladaptive perfectionism differentially predict enthusiastic and constrained commitment, which in turn influences EA. Our results showed that maladaptive perfectionism positively predicted constrained commitment (β = 0.70) and EA (β = 0.63), while negatively relating to enthusiastic commitment (β = −0.17). Conversely, adaptive perfectionism was positively associated with enthusiastic commitment (β = 0.24) and negatively with constrained commitment (β = −0.12). Moreover, enthusiastic commitment positively predicted EA (β = 0.24). We found a significant indirect effect between adaptive and maladaptive perfectionism when controlling for enthusiastic commitment, suggesting its dual role in this context. Our study suggests that enthusiastic commitment serves as a source of exercise addiction (EA) and has a dual role, acting as both a protective factor and a risk factor for it. Additionally, we found that maladaptive perfectionism is associated with higher levels of constrained commitment and EA, while correlating with lower levels of enthusiastic commitment. Conversely, adaptive perfectionism increases enthusiastic commitment and decreases constrained commitment. These findings highlight the associations between motivational and personality factors in EA, indicating that even adaptive traits can contribute to unhealthy exercise patterns in athletic environments.

## 1. Introduction

The concept of maintaining good health has become a social norm [1]. Regular exercise and a healthy diet are the most common ways to achieve this standard [2]. However, excessive focus on both can lead to addiction. For example, engaging in extreme amounts of physical activity may result in exercise addiction (EA), which could increase negative behavior such as eating disorders, psychological distress, insomnia, and body image concerns [3,4]. Athletes have a higher risk of EA than non-athletes. According to Juwono et al., the prevalence rate for athletes could be greater than 40% [5].

EA was initially defined by Baekeland in 1970 [6], but it was Glasser, in 1976 [7], who mistakenly characterized it as a positive addiction, believing it to have beneficial effects on health. However, this notion was swiftly debunked. Since then, a structured approach has emerged to identify EA, aligned with the seven criteria outlined in the DSM-5 (Diagnostic and Statistical Manual of Mental Disorders), adapted specifically for exercise behavior [8]. EA is recognized as a maladaptive phenomenon linked to the pursuit of regular moderate-to-vigorous exercise, mirroring the patterns observed in many other addictions, and it manifests through a range of deteriorative physiological and psychological effects [9,10]. EA is associated with higher levels of leptin and cortisol and has a negative impact on energy balance [11,12]. During periods of exercise deprivation, athletes who are exercise-addicted may experience decreased brain bioelectric activity, increased sympathetic activity, and heightened muscular tension [13]. Additionally, symptoms of anxiety, depression, and eating disorders are often linked to EA, which can impair cognitive function [14,15]. However, some studies suggest that EA can actually improve cognitive function, likely because it does not coexist with anxiety or depression in those cases [16,17].

The evolution of EA can be traced from the innocent joy of exercising for fun (‘want to’) to an escalating commitment that transforms it into a necessity (‘have to’). Ultimately, it becomes detached from enjoyment and performance goals, leading to an overwhelming compulsion [18,19]. McNamara and McCabe [20] used a biopsychosocial model to explain the predisposition, development, and maintenance of EA among athletes. This model encompasses various factors, including behavioral aspects (such as training frequency), psychological components (like pathological engagement, a lack of control, and preoccupation with body image), and physiological factors (e.g., addictive behaviors), as well as social influences (such as social isolation) and situational elements (e.g., high levels of competition and social pressure). Another model illustrates the interactions in EA [21]. This model suggests that EA depends on the interplay of personal factors (e.g., self-concept), situational influences (e.g., attractive alternatives), various exercise incentives (e.g., enjoyment, challenge), and exercise-related stressors (e.g., performance anxiety).

According to Marques et al. [22], the high prevalence of EA among athletes can be attributed to their increased levels of physical activity. Additionally, athletes often demonstrate heightened commitment and engagement due to their competitive nature, focus on achieving optimal sports performance, and the pressure to deliver results. Commitment seems to be important for EA. For example, several factors of the sport commitment model are included in the interaction model of EA [21]. Furthermore, previous studies have shown that commitment significantly influences exercise addiction [23]. However, commitment to exercise and EA are distinct concepts [24]. While exercise addicts are driven by internal compulsions, prioritize training above all else, and experience withdrawal when unable to exercise, committed athletes are motivated by external rewards, maintain a balanced perspective, and retain control over their training routines [25]. A key distinction lies in autonomy: committed athletes regulate their behavior, whereas exercise addicts feel controlled by their habits.

The concept of commitment was clearly defined by Scanlan et al. [26] in the sport commitment model, which distinguishes between two types of commitment. Enthusiastic commitment is described as a “psychological construct representing the desire and resolve to continue sports participation” [27] (p. 235). This type of commitment is characterized by an individual’s eagerness to participate in sports, driven primarily by sources such as enjoyment, opportunities, and personal investments [27]. Enthusiastic commitment is highly associated with intrinsic motivation; thus, enthusiastically committed athletes mainly participate in sports for internal reasons [28]. The other type is constrained commitment, defined as “the psychological construct representing perceptions of obligation to persist in a sport over time” [27] (p. 235). This type is characterized by the expectations and pressures that athletes feel while participating in a sport. The main sources of Constrained commitment include social constraints, supports, and competing priorities outside of sports. It is also associated with extrinsic motivational factors [28]. The significance of each commitment type is influenced by various factors, including the nature of the sport (individual vs. team sports), level of competition, and gender. For example, enthusiastic commitment is often more prevalent among participants in team sports. Additionally, the level of competition plays a crucial role: as the competition level increases, so does motivation and enthusiastic commitment. However, this increased competition can also lead to stronger feelings of compulsion, driving athletes toward constrained commitment [29]. There are gender differences in commitment research as well. Generally, men are more commitment-driven than women, but there are some inconsistences in the literature [30,31].

In the view of individual psychological factors, perfectionism significantly influences an athlete’s commitment to sport. This trait often stems from excessively high personal expectations and an overly critical self-esteem [32]. Research by Jowett et al. [33] and Larkin et al. [34] has found that perfectionism can enhance athletes’ dedication to their sport. Additionally, Vink and Raudsepp [35] reported a one-way relationship between perfectionism and commitment to sport-specific activities among youth athletes in team sports. As a personality trait, perfectionism plays a crucial role in driving competitive spirit, the desire to improve, and the determination to win [36]. Performance and perfectionism often coexist; however, while perfectionism is generally regarded as a dysfunctional trait in many areas of psychology, it can lead to negative emotional states, anxiety, depression, maladaptive behaviors, and harmful addictions [37,38]. It is also frequently associated with eating disorders and body image issues [39,40,41]. Moreover, perfectionism can adversely affect performance. High levels of performance anxiety can undermine self-confidence and create a fear of making mistakes, resulting in significant stress [42]. Thus, perfectionism is a dual phenomenon encompassing both adaptive and maladaptive forms [43,44]. Adaptive perfectionism is characterized by realistic and reasonable expectations based on self-awareness, allowing individuals to recognize their strengths and limitations. In contrast, maladaptive perfectionism involves unrealistic expectations, the setting of unattainable goals, and an intense fear of making mistakes.

Among the various personality traits that potentially influence EA, perfectionism stands out as the most significant [45]. Several researchers have explored the relationship between EA and perfectionism, concluding that perfectionism acts as a precursor to EA [46,47,48,49,50]. Maladaptive perfectionism has been specifically linked to EA; however, adaptive perfectionism has not been associated with it [51,52]. This contrast can be found when the relationship between commitment and perfectionism is explored. For example, a previous study identified two types of perfectionists: positive and negative. Positive perfectionists are characterized by participating in sports for internal reasons, such as enjoyment and enthusiastic commitment, while negative perfectionists are driven by constrained reasons, including constrained commitment [53].

As noted, a number of researchers have examined the relationships between perfectionism, sport commitment, and EA. However, the connections among all three of these concepts, when assessed within the same sample and at the same time, remain unexplored. Therefore, our objective was to investigate the associations between perfectionism, commitment, and EA among young Hungarian athletes participating in various sports. Athletes in Hungary are an ideal population for examining the links between perfectionism, commitment, and EA due to their high achievement orientation and intense training demands [54]. They often display elevated levels of both adaptive and maladaptive perfectionism, which makes them particularly relevant for understanding how these traits relate to motivational patterns and potentially harmful behaviors [55]. Their structured routines and strong goal orientation provide a clear context for assessing how different types of commitment contribute to the risk of EA. Moreover, insights gained from athletes may also apply to other populations as well.

**Figure 1:**
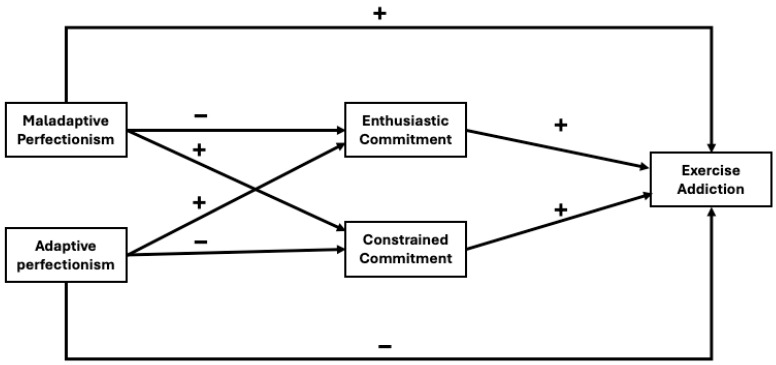
The hypothesized associations between perfectionism, commitment, and exercise addiction.

Based on previous research, our goal is to investigate the relationships among perfectionism, sport commitment, and EA. We hypothesized a theoretical model (Figure 1). We hypothesized that adaptive perfectionism would be positively associated with enthusiastic commitment and negatively associated with constrained commitment and EA, while maladaptive perfectionism would be positively associated with constrained commitment and EA and negatively associated with enthusiastic commitment. Furthermore, we also hypothesized a positive relationship between EA and enthusiastic and constrained commitment.

## 2. Materials and Methods

### 2.1. Participants and Procedure

The study involved a sample of 219 young adults aged between 18 and 25 years, consisting of 107 men and 112 women. The average age of participants was 22.19 years, with a standard deviation of 1.74. Participants were recruited from various sports, including aikido, ice hockey, football, basketball, and athletics. Details on sport type frequency can be found in the Appendix Table A1. In total, 121 individuals participated in individual sports, while 98 were involved in team sports. The study included amateur athletes who had been members of a sports club for an average of 7.5 years and were competing at the national level. Convenience sampling was used for data collection; hence, they were recruited via online forums (e.g., Facebook) and were asked to fill out our questionnaire. It took approximately 10 min to complete. Prior to data collection, the participants were informed about the study and assured that their participation was voluntary and anonymous, with no personal information, such as names, collected. All participants also provided online informed consent to partake in the study. The research received ethical approval from the Institutional Review Board of the Hungarian University of Sport Science (Ethical Approval Number: MTSE-OKE-KEB/03/2023).

### 2.2. Measures

#### 2.2.1. Multidimensional Perfectionism Scale

Perfectionism was assessed using Frost’s Multidimensional Perfectionism Scale [56], which has been previously translated and adapted for the Hungarian and sport context [57,58]. The scale consisted of 35 items divided into six subscales. Four of these subscales were associated with maladaptive perfectionism: parental expectation, parental criticism, doubt about action, and concern over mistakes. The remaining two subscales reflected adaptive perfectionism: personal standards and organization. The answer categories were presented on a 5-point Likert-type scale (1 = Strongly disagree; 5 = Strongly agree). The Cronbach’s alpha for the six subscales ranged from 0.76 to 0.92. For the purposes of this study, only the adaptive and maladaptive forms of perfectionism were analyzed.

#### 2.2.2. Sport Commitment Questionnaire

Enthusiastic and constrained types of sport commitment were assessed using the subscales of Scanlan’s Sport Commitment Questionnaire-2 [27]. This scale has been previously validated and used in Hungarian contexts [31]. Enthusiastic commitment was measured using six items (e.g., “I am dedicated to continuing to play this sport”; [27] (p. 245)), while constrained commitment was assessed with five items (e.g., “Staying in this sport is more of a necessity than a desire”; [27] (p. 245)). Participants responded on a 5-point Likert-type scale, where 1 indicated “strongly disagree” and 5 indicated “strongly agree.” The internal reliability, measured by Cronbach’s alpha, was 0.78 for enthusiastic commitment and 0.81 for constrained commitment.

#### 2.2.3. Exercise Addiction Inventory

The Hungarian version of the Exercise Addiction Inventory (EAI) was used to assess the risk of exercise addiction [59,60]. It included six items that show the key aspects of exercise behaviors: (1) salience (importance of exercise in daily life), (2) mood modification (exercise’s role in altering mood), (3) tolerance (need for increased exercise frequency or intensity), (4) withdrawal symptoms (negative effects when exercise is unavailable), (5) conflict (exercise-related interference in social or personal areas), and (6) relapse (tendency to return to previous levels of exercise after periods of reduced activity). All answer categories were on a five-point Likert type scale (1 = strongly disagree; 5 = strongly agree). Scores for each item were summed, with the total score ranging from 6 to 30. Higher scores suggested a greater risk of exercise addiction; however, means were used in this study to help comparisons with other measures. It is important to emphasize that the EAI is not a diagnostic tool but rather a validated screening instrument designed for the efficient identification of individuals at risk of exercise addiction [61]. The Cronbach alpha value was 0.81 in this study.

### 2.3. Statistical Analysis

Skewness and kurtosis values ranged from −1.5 to 1.5, indicating that our variables can be considered normally distributed, in line with Forero’s recommendations [62]. Consequently, we employed independent sample *t*-tests and Pearson’s correlation for our primary analysis. An independent samples *t*-test was conducted to investigate potential gender differences, utilizing Cohen’s d as the effect size. Additionally, correlation analysis was performed to examine the bivariate relationships among the study variables: maladaptive perfectionism, adaptive perfectionism, enthusiastic commitment, constrained commitment, and exercise addiction. For our main analysis, we performed path analysis to test the hypothesized model. We assessed model fit using multiple indices, including the chi-square (χ^2^) test, the relative chi-square divided by degrees of freedom (CMIN/d.f.), the comparative fit index (CFI), the Tucker–Lewis index (TLI), the standardized root mean square residual (SRMR), and the root mean square error of approximation (RMSEA). The acceptable values for these indices were as follows: CMIN/d.f. < 3.0; RMSEA < 0.08; TLI > 0.90; CFI > 0.90; and SRMR < 0.08 [63,64,65,66]. Indirect pathways were calculated using 95% confidence intervals (CIs) to assess the presence and significance of mediating effects within the model. All analyses were conducted at a significance level of *p* < 0.05. Jamovi 2.6 was used for data analysis, which is an open-source software and was freely accessed.

## 3. Results

Table 1 presents the descriptive statistics and gender differences among the study variables. We found significant differences only in adaptive perfectionism (*p* < 0.05). The effect sizes indicated small effects in most cases, with the exception of adaptive perfectionism. Given that there was only one significant difference, we decided not to include gender in further analyses. Correlation analysis (Table 2) revealed significant linear relationships in most of the study variables. However, maladaptive perfectionism did not significantly correlate with enthusiastic commitment (r = −0.06), and the two types of commitment did not show any association with each other (r = −0.02).

**Figure 2:**
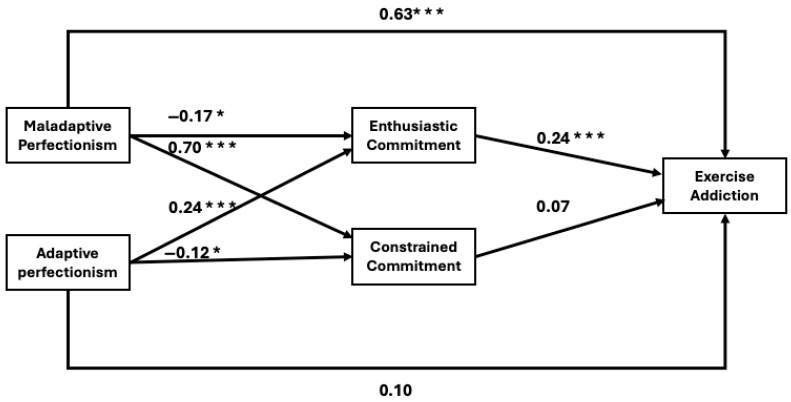
The direct effects of perfectionism, commitment, and exercise addiction. **Note:** * *p* < 0.05; *** *p* < 0.001.

In the next part of our study, we employed path analysis to test our hypothesized model (Figure 2). The results indicated a strong model fit (χ^2^(2) = 4.16; CMIN/df = 2.08; SRMR = 0.02; RMSEA = 0.07; CFI = 0.99; TLI = 0.97), suggesting that our model is well-established and that the variables follow the expected theoretical order. The analysis revealed that maladaptive perfectionism significantly and positively predicts constrained commitment (β = 0.70) and EA (β = 0.63), while negatively predicting enthusiastic commitment (β = −0.17). In contrast, adaptive perfectionism showed a negative association with constrained commitment (β = −0.12) and a positive association with enthusiastic commitment (β = 0.24). Additionally, enthusiastic commitment positively predicted EA (β = 0.24) within this sample.

The indirect and total effects, along with a 95% confidence interval, are presented in Table 3. Maladaptive perfectionism had an indirect effect on EA through enthusiastic commitment (β = −0.04). In contrast, adaptive perfectionism also had a significant indirect effect through enthusiastic commitment (β = 0.06).

## 4. Discussion

This study examined the relationships between perfectionism, commitment, and EA among Hungarian athletes. Adaptive perfectionism was hypothesized to relate positively to enthusiastic commitment and negatively to constrained commitment and EA, while maladaptive perfectionism was expected to show the opposite pattern. The results showed that maladaptive perfectionism positively predicted constrained commitment and EA and negatively predicted enthusiastic commitment. Adaptive perfectionism showed the reverse pattern. Additionally, enthusiastic commitment positively predicted EA.

A direct effect was observed between maladaptive perfectionism and exercise addiction, which is not surprising given that exercise addiction is generally considered a maladaptive outcome [9,10]. Maladaptive perfectionism in athletes often leads to dissatisfaction with performance and increased anxiety, which can contribute to exercise addiction as athletes overcompensate for perceived inadequacies [67]. A previous study highlighted that this risk is particularly higher for high-intensity sports like CrossFit and running, since it requires significant physical effort and commitment, which can lead to increased pressure on athletes to perform well [49]. Furthermore, in competitive environments like CrossFit and running, athletes frequently compare themselves to others. This can exacerbate feelings of inadequacy and lead to a heightened focus on perfectionism, as they strive to meet or exceed the standards set by peers [49]. While our sample did not include CrossFit and running athletes, it still contained several sports that require higher intensity for performance. For example, we had representatives from bodybuilders (*n* = 7), wrestlers (*n* = 14), karate practitioners (*n* = 8), and Thai boxers (*n* = 3), among others, contributing to a substantial sample size.

The relationship between perfectionism and the two forms of commitment aligned well with the existing literature [53]. Studying perfectionism is important among various background factors, as it represents a somewhat contradictory personality trait. On the one hand, the pursuit of perfection can foster motivation for personal growth, a strong orientation toward success, and enhanced performance [32,36]. On the other hand, it can make individuals more susceptible to anxiety, adjustment disorders, and harmful substance use, which may hinder their performance [37,38,42]. Our data support this duality when examining the associations between the two types of commitment and the two forms of perfectionism. The reason behind our results is that adaptive perfectionism is associated with internal motivations, such as enjoyment, while maladaptive perfectionism is driven by external pressures.

There were some unexpected results in our analysis. We found indirect effects between both types of perfectionism and EA, mediated by enthusiastic commitment, indicating that enthusiastic commitment has a dual role, acting both as a risk factor and a protective factor for EA depending on what kind of perfectionism it is connected to. Furthermore, enthusiastic commitment was also found to have positive effect on EA in a direct path, which was also unexpected and contrary to our initial hypotheses. Adaptive perfectionism with enthusiastic commitment appears to function as a risk factor for EA. Individuals high in adaptive perfectionism are driven by intrinsic motivation and a genuine enjoyment of exercise. As a result, their enthusiastic commitment may lead to increased exercise engagement, which, while typically healthy, can cross into addictive behavior when exercise becomes excessive or starts interfering with other areas of life. In this context, enthusiastic commitment amplifies the risk of EA by reinforcing high levels of engagement, even when it becomes compulsive. This finding contradicts previous studies, which suggested that intrinsic factors have negative effects on EA [68]. We believe these results indicate that the behavior behind enthusiastic commitment can closely resemble addiction among athletes, which can evolve overtime, since individuals may increase their exercise amounts and experience withdrawal symptoms, euphoria, and relapse, which can lead to detrimental effects on their social lives and overall health [69].

In contrast to these results, enthusiastic commitment, when associated with maladaptive perfectionism, has a protective role. Maladaptive perfectionism is generally characterized by self-critical tendencies and a fear of failure, which are often accompanied by lower levels of intrinsic motivation. For individuals with high levels of maladaptive perfectionism, the presence of enthusiastic commitment may buffer against the compulsive and rigid nature of their exercise behavior by introducing a degree of enjoyment and autonomy. Thus, enthusiastic commitment in this context can mitigate the negative impact of maladaptive perfectionism on EA. These findings suggest that enthusiastic commitment is not unilaterally adaptive or maladaptive. Rather, its influence on EA appears to be context-dependent, shaped by the underlying motivational patterns associated with different forms of perfectionism. This highlights the importance of considering the quality of motivation when evaluating risk factors for exercise addiction.

Interestingly, neither adaptive perfectionism nor constrained commitment showed a significant direct effect on EA as it was hypothesized earlier. This may be explained by the underlying nature of these constructs. Adaptive perfectionism is typically characterized by high personal standards, goal-directed behavior, and emotional regulation. While individuals with high adaptive perfectionism are highly engaged and motivated, they also tend to possess psychological flexibility and self-acceptance, which may protect them from developing compulsive behaviors. Therefore, although adaptive perfectionism was found to indirectly increase exercise addiction through enthusiastic commitment, it does not appear to exert a direct influence on addictive tendencies. The positive motivational qualities associated with adaptive perfectionism may buffer against the risk of excessive, maladaptive exercise behavior. Similarly, constrained commitment (reflecting obligations, pressure, or a sense of duty) did not significantly predict exercise addiction directly. One possible explanation is that individuals who exercise out of obligation or external pressure may not engage in exercise with the frequency or intensity required to reach the threshold of addiction. Moreover, constrained commitment may result in ambivalent or inconsistent exercise behavior, which does not align with the persistent, high-intensity patterns often seen in exercise addiction. Thus, while constrained commitment might contribute to stress or dissatisfaction, it may not serve as a strong, direct driver of exercise addiction.

In summary, our findings supported our hypotheses regarding the relationships between the two types of perfectionism, the two forms of commitment, and maladaptive perfectionism’s link to exercise addiction. However, our expectations were not confirmed in the case of adaptive perfectionism, particularly in relation to both forms of commitment and EA. Additionally, the mediating role of enthusiastic commitment was unexpected, as it appeared to function as both a protective and a risk factor for EA.

Irrespective of our findings, the study has some limitations that need to be addressed. First, while we used the EAI as a screening tool, it is important to note that exercise addiction currently lacks formal diagnostic criteria in the DSM-5. The EAI identifies risk rather than clinical diagnosis, and true prevalence rates may differ from questionnaire-based risk assessments. Furthermore, generalizability is limited because of convenience sampling, and data collection utilizing social media platforms may have narrowed down participation. We had some limitations in our results as well. For example, the indirect effects were small, besides the significant paths. Additionally, we only investigated adaptive and maladaptive forms of perfectionism, and in future studies, we could examine different subtypes of perfectionism. This applies to factors of sport commitment as well, which could provide other potential mediators for EA. In the future, we aim to investigate the role of these constructs more deeply by performing analyses with different types of sports, since, as has been noted, there are sports that have increased levels of EA. Besides these, we believe our results provide useful insights into the roles of EA, perfectionism, and commitment among athletes.

## 5. Conclusions

Our findings emphasize the significance of motivational quality in the development of exercise addiction. Based on our results, we can draw the following conclusions: (1) Enthusiastic commitment plays a dual role, serving as both a protective factor and a risk factor for EA. (2) Enthusiastic commitment could be a source for EA. (3) Maladaptive perfectionism is linked to higher levels of constrained commitment and EA, while it is associated with lower levels of enthusiastic commitment. (4) In contrast, adaptive perfectionism promotes enthusiastic commitment and reduces constrained commitment, but it does not directly predict EA. These results illustrate the context-dependent role of motivational factors in EA and suggest that even adaptive traits may have unintended consequences in sport environments.

## Figures and Tables

**Table 1:** Characteristics and gender differences for the observed variables.

Variable	Total (M; SD)	Men (M; SD)	Women (M; SD)	t-Value	Cohen’s d
**Maladaptive Perfectionism**	3.32 (0.96)	3.35 (0.93)	3.29 (1.00)	−0.40	0.05
**Adaptive Perfectionism**	3.93 (0.57)	4.01 (0.56)	3.87 (0.56)	−2.04 *	0.28
**Enthusiastic Commitment**	3.99 (0.70)	4.05 (0.67)	3.93 (0.73)	−1.22	0.16
**Constrained Commitment**	3.36 (1.02)	3.36 (0.97)	3.36 (1.07)	−0.03	0.00
**Exercise Addiction**	3.68 (0.85)	3.69 (0.86)	3.69 (0.86)	0.21	0.03

Note. * *p* < 0.05.

**Table 2:** Correlation analysis of the observed variables.

Variable	1	2	3	4
**1. Maladaptive Perfectionism**	—			
**2. Adaptive Perfectionism**	0.45 ***	—		
**3. Enthusiastic Commitment**	−0.06	0.17 *	—	
**4. Constrained Commitment**	0.64 ***	0.19 **	−0.02	
**5. Exercise Addiction**	0.70 ***	0.43 ***	0.22 **	0.48 ***

Note. * *p* < 0.05; ** *p* < 0.01; *** *p* < 0.001.

**Table 3:** Indirect and total effects of the model.

Dependent Variable	Model Path	Coefficient (β)	SE	Lower CI (95%)	Upper CI (95%)
Exercise Addiction	Total effects				
	AP → EA	0.15 **	0.08	0.45	0.62
	Total effects				
	MP → EA	0.63 ***	0.05	0.04	0.45
	Indirect effects				
	AP → EC → EA	0.06 **	0.03	0.02	0.09
	Indirect effects				
	AP → CC → EA	−0.01	0.01	−0.02	0.09
	Indirect effects				
	MP → EC → EA	−0.04 *	0.02	−0.07	0.02
	Indirect effects				
	MP → CC → EA	0.05	0.04	−0.03	0.11

Note: * *p* < 0.05; ** *p* < 0.01; *** *p* < 0.001. EA = Exercise addiction; AP = adaptive perfectionism; MP = maladaptive perfectionism; EC = enthusiastic commitment; CC = constrained commitment. CIs were calculated using the bootstrap method.

## Data Availability

The data presented in this study are available on request from the corresponding author. The data are not publicly available due to privacy reasons.

## Abbreviations

The following abbreviations are used in this manuscript:

EA	Exercise addiction
EAI	Exercise addiction Inventory
CFI	Comparative fit index
TLI	Tucker–Lewis index
RMSEA	Root mean square error of approximation
SRMR	Standardized root mean square residual
CMIN/df.	Relative chi-square divided by the degrees of freedom

## Appendix A

**Table A1:** Sport type frequencies in our sample.

Sport	Frequency	Percentage of Total (%)
Aikido	1	0.0
American Football	1	0.0
Athletics	4	2.0
Wrestling	14	6.0
Boxing	3	1.0
Fin swimming	1	0.0
Powerlifting	1	0.0
Rowing	21	10.0
Running	1	0.0
Judo	7	3.0
Ice Hockey	10	5.0
Karate	8	4.0
Cycling	1	0.0
Classical Ballet	4	2.0
Basketball	15	7.0
Handball	22	10.0
Football (Soccer)	33	15.0
Horseback Riding	1	0.0
Volleyball	11	5.0
Shooting Sports	1	0.0
Artistic Gymnastics	1	0.0
Taekwondo	1	0.0
Tennis	2	1.0
Bodybuilding	7	3.0
Thai Boxing	3	1.0
Triathlon	8	4.0
Dance	8	4.0
Jump Rope	3	1.0
Water Polo	6	3.0
Fencing	1	0.0
Archery	5	2.0
Swimming	7	3.0
Kayak-Canoe	7	3.0
